# Transcriptome analysis by GeneTrail revealed regulation of functional categories in response to alterations of iron homeostasis in *Arabidopsis thaliana*

**DOI:** 10.1186/1471-2229-11-87

**Published:** 2011-05-18

**Authors:** Mara Schuler, Andreas Keller, Christina Backes, Katrin Philippar, Hans-Peter Lenhof, Petra Bauer

**Affiliations:** 1 Dept. of Biosciences - Botany, Campus A2.4, Saarland University, D-66123 Saarbrücken, Germany; 2 Dept. of Informatics - Center for Bioinformatics, Campus E1.1, Saarland University, D-66123 Saarbrücken, Germanys; 3 Dept. Biology I - Plant Biochemistry and Physiology, Biocenter of the Ludwig-Maximilians-University München, Großhadernerstr. 2-4, D-82152 Planegg-Martinsried, Germany

## Abstract

**Background:**

High-throughput technologies have opened new avenues to study biological processes and pathways. The interpretation of the immense amount of data sets generated nowadays needs to be facilitated in order to enable biologists to identify complex gene networks and functional pathways. To cope with this task multiple computer-based programs have been developed. GeneTrail is a freely available online tool that screens comparative transcriptomic data for differentially regulated functional categories and biological pathways extracted from common data bases like KEGG, Gene Ontology (GO), TRANSPATH and TRANSFAC. Additionally, GeneTrail offers a feature that allows screening of individually defined biological categories that are relevant for the respective research topic.

**Results:**

We have set up GeneTrail for the use of *Arabidopsis thaliana*. To test the functionality of this tool for plant analysis, we generated transcriptome data of root and leaf responses to Fe deficiency and the Arabidopsis metal homeostasis mutant *nas4x-1*. We performed Gene Set Enrichment Analysis (GSEA) with eight meaningful pairwise comparisons of transcriptome data sets. We were able to uncover several functional pathways including metal homeostasis that were affected in our experimental situations. Representation of the differentially regulated functional categories in Venn diagrams uncovered regulatory networks at the level of whole functional pathways. Over-Representation Analysis (ORA) of differentially regulated genes identified in pairwise comparisons revealed specific functional plant physiological categories as major targets upon Fe deficiency and in *nas4x-1*.

**Conclusion:**

Here, we obtained supporting evidence, that the *nas4x-1 *mutant was defective in metal homeostasis. It was confirmed that *nas4x-1 *showed Fe deficiency in roots and signs of Fe deficiency and Fe sufficiency in leaves. Besides metal homeostasis, biotic stress, root carbohydrate, leaf photosystem and specific cell biological categories were discovered as main targets for regulated changes in response to - Fe and *nas4x-1*. Among 258 differentially expressed genes in response to - Fe and *nas4x-1 *five functional categories were enriched covering metal homeostasis, redox regulation, cell division and histone acetylation. We proved that GeneTrail offers a flexible and user-adapted way to identify functional categories in large-scale plant transcriptome data sets. The distinguished feature that allowed analysis of individually assembled functional categories facilitated the study of the *Arabidopsis thaliana *transcriptome.

## Background

High-throughput technologies for transcriptional profiling have strongly advanced our understanding of complex networks of gene interactions in physiology and development. The most common integrative approach for measuring gene expression is microarray analysis, which has already been applied to investigate many biological processes. For storing the vast amount of measured expression profiles, many freely available repositories have been developed, including the Gene Expression Omnibus (GEO) [1] or Stanford Microarray Database (SMD) [2]. It has become a routine habit for many researchers to consult published microarray expression data for theoretical modeling of regulatory networks involving their favourite genes prior to experimentation [3,4]. The full strength of microarray interpretation lies in the possibility of extracting information beyond the single gene level to address questions on the co-regulation of genes, on the identification of gene networks and entire extensive pathways of genes acting in the same physiological process. Specialized software tools like Genevestigator [4], the Botany Array Resource (BAR) [5], MapMan [6], ATTED-II [7,8] or VirtualPlant [9] for example have been developed to answer such complex questions in plants.

The analysis software tool GeneTrail [10] can be used for comparative analysis of transcriptome data to identify functional clusters or pathways rather than single genes that are affected in one experimental condition compared to another. This user-friendly and freely available tool covers analysis of a wide spectrum of available biological categories assembled from information of the Kyoto Encyclopedia of Genes and Genomes (KEGG), Gene Ontology (GO), TRANSPATH pathways and transcription factors from TRANSFAC. An advantage of GeneTrail is that functional categories for investigation by the program need not to be predefined by the software developers, the categories can also be created by the users themselves according to their personal fields of interest. Therefore, the GeneTrail tool allows individual users a flexible pathway analysis when comparing two different samples.

GeneTrail has already been applied to analyse transcriptome data of a wide range of model organisms including *Homo sapiens *and *Mus musculus *[11-13]. Here, we demonstrate the functionality of GeneTrail for plant transcriptome analysis beyond the single gene level.

Our example of application was based on the comparisons of the root and leaf transcriptomes of the metal homeostasis mutant *nas4x-1 *[14] and wild type plants in response to sufficient and deficient Fe supply. Our study focused on the regulatory patterns of entire response pathways. These response pathways included cellular categories derived from KEGG, GO, TRANSPATH and TRANSFAC, plant-specific response pathways described in MapMan [6] and an individually assembled category named "metal homeostasis". Gene Set Enrichment Analysis (GSEA) of all genes and Over-Representation Analysis (ORA) of the selected differentially expressed genes provided complex information on regulatory networks at the level of gene categories and pathways.

## Methods

### Plant material and growth conditions

The *nas4x-1 *mutant plant line used has been described in [14]. Wild type and *nas4x-1 *plants were grown in a hydroponic solution containing a quarter strength of Hoagland salts (0.1875 mM MgSO_4 _× 7 H_2_O, 0.125 mM KH_2_PO_4_, 0.3125 mM KNO_3_, 0.375 mM Ca(NO_3_)_2_, 12.5 μM KCL, 12.5 μM H_3_BO_3_, 2.5 μM MnSO_4 _× H_2_O, 0.5 μM ZnSO_4 _× 7 H_2_O, 0.375 μM CuSO_4 _× 5 H_2_O, 0.01875 μM (NH_4_)_6_Mo_7_O_24 _× 4 H_2_O, pH 6.0) supplied with 10 μM FeNa-EDTA. The medium was exchanged weekly. Four weeks after germination, plants were exposed for another week to plant medium containing either 10 μM FeNa-EDTA (+ Fe) or without Fe (- Fe). Cultivation took place at 21°C/19°C and 16 h light, 8 h dark cycles and a light intensity of 150 μmol × m^-2 ^× s^-1^.

### RNA extraction and microarray hybridization

L3/ L4 rosette leaves and roots of wild type and *nas4x-1 *mutant plants grown under + and - Fe were harvested separately in liquid nitrogen (total of 8 samples). Experiments were performed three times in three consecutive weeks and respective samples were harvested to obtain 3 biological replicates (n = 3; Additional file 1, Figure S1A). Total RNA was extracted from 100 mg of root or leaf material with the Qiagen RNeasy Plant Mini Prep Kit according to the manufacturer's protocol. 5 μg RNA were processed into biotin-labeled cRNA and hybridized to Affymetrix GeneChip Arabidopsis ATH1 Genome Arrays (Affymetrix, High Wycombe, U.K.), using the Affymetrix One-Cycle Labeling and Control (Target) kit according to the manufacturer's instructions. Microarray signals were determined using Affymetrix Microarray Suite 5.1.(MAS 5.1) and made comparable by scaling the average overall signal intensity of all probe sets to a target signal of 100 (Affymetrix GeneChip Operating software, GCOS) [15,16]. Data are available under http://www.ncbi.nlm.nih.gov/geo/query/acc.cgi?acc=GSE24348.

### Statistical analysis of microarray expression data and calculation of fold changes

For further data analysis, the data extracted from the Affymetrix Microarray Suite Microarray were processed by using standard quantile normalization [17], which has become one of the most commonly used normalization techniques for microarray data and finds also application in pre-processing packages as e.g., the "Robust Multichip Average"(RMA) approach [18]. Median values were calculated from the normalized expression signals of the three biological replicates. Fold changes were calculated from median values for eight comparisons of the eight data sets, namely - Fe vs. + Fe (WT), - Fe vs. + Fe (*nas4x-1), nas4x-1 *vs. WT (+ Fe), *nas4x-1 *vs. WT (- Fe), for roots and leaves, respectively (see Additional file 1, Figure S1D).

### GeneTrail

The web-based application GeneTrail [10,19] provided two basic approaches for assessing the enrichment or depletion of gene sets: the unweighted Gene Set Enrichment Analysis (GSEA) and the Over-Representation Analysis (ORA).

GeneTrail supported a variant of unweighted GSEA [20]. The input for a GSEA was a list of genes or proteins that were sorted by an arbitrary criterion (e.g., fold changes of expression values). For computing the statistical significance of a biological category, a Kolmogorov-Smirnov-like test was used that computed whether the genes in the category were equally distributed (category was not enriched) or accumulated on top (see example in Additional file 2, Figure S2A) or on bottom (see example in Additional file 2, Figure S2B) of the list. To this end, a running sum was computed as follows: When processing the input list from top to bottom, the running sum was increased each time a gene belonged to the biological category, otherwise the running sum was decreased. Red graphs with a 'mountain-like shape' illustrated a specific category predominantly containing top-ranked genes (see example in Additional file 2, Figure S2A). In contrast, green graphs with a 'valley-like shape' illustrated a specific category predominantly containing bottom-ranked genes (see example in Additional file 2, Figure S2B). The enrichment of a category did not imply a differential expression of all genes of this category. The expression values of every single gene were interpreted and evaluated individually. For estimating the statistical significance, the maximal deviation from zero of the running sum was considered. If this maximal deviation was positive, the category was enriched for the test set genes, otherwise it was depleted. In GeneTrail, the p-value was computed as the probability that any running sum reached a larger or equal absolute maximal deviation from zero. To perform GSEA fold changes were generated to compare two samples, which were then sorted according to values from highest to lowest. Sorted gene identifiers were uploaded as text file prior to performing GSEA.

An ORA compared a set of interesting genes or proteins (test set) to a background distribution (reference set) concerning a certain biological category (e.g. a metabolic pathway). The distribution of test set genes that were contained in the considered biological category were compared to the genes of the reference set having this property. If more genes in the test set belonged to the considered biological category than expected, this category was enriched or over-represented, otherwise the category was depleted or under-represented in the test set. In GeneTrail, the statistical significance was assessed by computing a one-tailed p-value using the hypergeometric distribution.

If not mentioned otherwise, we performed all analyses with GeneTrail using the following parameters: p-value adjustment: FDR, significance threshold: 0.05. The number of two genes per category was set as minimum number for all analyses. As reference set for performing an ORA, we used all genes present on the ATH1 chip. All analysis results computed with GeneTrail are available on the web-site http://genetrail.bioinf.uni-sb.de/paper/ath/, where links to GSEA and ORA results are provided (The original GeneTrail results pages can be accessed under the file named SummaryPage.html for all comparisons).

### NIA Array Analysis Tool

For statistical treatment and identification of differentially expressed genes from pairwise comparisons, the web-based software NIA Array Analysis tool developed by the National Institute on Aging [21] was utilized. The statistical analysis performed with this online tool was based on the single-factor ANalysis Of VAriance (ANOVA). The statistical significance was determined using the False Discovery Rate (FDR) method. The data were statistically analyzed using the following settings: error model ´max (average, actual)', 0.01 proportion of highest variance values to be removed before variance averaging, 10 degrees of freedom for the Bayesian error model, 0.05 Benjamini and Hochberg False discovery rate (FDR) threshold, zero mutations.

## Results

### Adaptation of GeneTrail for the use of *Arabidopsis thaliana*

In order to utilize GeneTrail for *Arabidopsis thaliana*, we extended GeneTrail such that, besides our supported default identifiers, Arabidopsis-specific identifiers (AGI gene codes from TAIR, transcript IDs from the ATH1 microarray) could be used. In addition, we allowed for the usage of the ATH1 chip as pre-defined reference set. Moreover, we improved the handling of individually defined categories. As default analyses for Arabidopsis, we included KEGG, GO, Homologene, and the search for an arbitrary amino acid sequence motif.

### Experimental design

In order to evaluate the GeneTrail tool for plant-specific analysis, we generated and used transcriptome data sets of *nas4x-1 *mutants compared to wild type plants grown under + and - Fe supply (Additional file 1, Figure S1). The quadruple *nas4x-1 *mutant harbours T-DNA insertions in the four *NICOTIANAMINE SYNTHASE (NAS) *genes present in the Arabidopsis genome. In consequence this mutant shows a strongly reduced nicotianamine (NA) level [14]. Since nicotianamine acts as chelator for Fe, Cu and Zn, *nas4x-1 *mutants have a defect in transport and allocation of these metals throughout the plant [14]. Microarray experiments were conducted using the Arabidopsis ATH1 GenChip (Affymetrix). For this study, four-week old *nas4x-1 *mutant and wild type plants were exposed for 7 days to + and - Fe supply. These conditions have been established previously and have resulted in a reproducibly strong interveinal leaf chlorosis of *nas4x-1 *plants compared to wild type, especially upon Fe deficiency conditions (Additional file 1, Figure S1B) [14]. The experiment was repeated three times in consecutive weeks to obtain three independent biological repetitions. Rosette leaves and roots of five week-old plants were harvested and microarray hybridization experiments were performed. Normalized expression values (available from GEO under http://www.ncbi.nlm.nih.gov/geo/query/acc.cgi?acc=GSE24348) were either processed and further analysed in GeneTrail or screened for differentially expressed genes with the NIA array tool and subsequently used for GeneTrail (see experimental outline in Additional file 1, Figure S1A, S1C). A total of eight meaningful pair-wise comparisons between the eight data sets was considered in our analysis, namely - Fe vs. + Fe (WT), - Fe vs. + Fe (*nas4x-1), nas4x-1 *vs. WT (+ Fe), *nas4x-1 *vs. WT (- Fe), for roots and leaves, respectively (Additional file 1, Figure S1D).

### Gene Set Enrichment Analysis (GSEA) using general biochemical and cell biological categories from KEGG, TRANSPATH, GO and TRANSFAC

To identify functional categories that were significantly differentially regulated between *nas4x-1 *and wild type and between + and - Fe samples we performed Gene Set Enrichment Analysis (GSEA). GeneTrail-predefined categories from KEGG, TRANSPATH, GO and TRANSFAC were used in GSEA for the eight pair-wise comparisons that were mentioned in the previous paragraph to be meaningful to us (see also Additional file 1, Figure S1D). Comparing - Fe vs. + Fe in wild type we could identify nine induced categories belonging to four different areas (carbohydrate and energy, oxidoreductase activity, defense response, nitrate and amino acid metabolism), and 17 repressed categories belonging to 11 different areas (dolichol metabolism, cold response, prenol metabolism, chloroplast, flavonoid metabolism, nucleoside metabolism, COP1, cellulose activity, fatty acid metabolism, phototropism, DNA polymerase) (Tables 1 and Additional file 3, Table S1). When comparing *nas4x-1 *samples, - Fe vs. + Fe, we identified five categories of three different areas (Fe transport, protease, secondary metabolism) that were induced, whereas three categories of two different areas (hormone/auxin transport, tubulin) were repressed (Tables 1 and Additional file 3, Table S1). When comparing + Fe samples, *nas4x-1 *vs. wild type, we found that 16 categories of five different areas (pyrimidin metabolism, nutrient reservoir, metal homeostasis, defense/glucosinolate/chitinase, general metabolism) were induced while five categories of three different areas (sucrose, fatty acid, protein synthesis) were repressed (Tables 1 and Additional file 3, Table S1). Finally in the comparison of - Fe samples, *nas4x-1 *vs. wild type, only five categories of two different areas (metal, ATPase) were induced, and no categories were found repressed (Tables 1 and Additional file 3, Table S1). From these data we can conclude that the number of differentially regulated categories was highest in the comparisons of wild type - Fe vs. + Fe (in total 26 categories belonging to 15 areas, Tables 1 and Additional file 3, Table S1) and of + Fe, *nas4x-1 *vs. wild type (in total 21 categories belonging to eight areas, Tables 1 and Additional file 3, Table S1) suggesting that cellular physiology of the plants from which the samples had been taken had been drastically affected by the treatment (wild type + vs. - Fe) and by the mutation (+ Fe *nas4x-1 *vs. wild type). On the other other hand, the number of differentially regulated categories was low when comparing *nas4x-1 *samples with each other (in total eight categories belonging to five areas, Tables 1 and Additional file 3, Table S1) and *nas4x-1 *with wild type at - Fe (in total five categories belonging to two areas, Tables 1 and Additional file 3, Table S1). The latter observation suggests that few cell physiological changes had occurred between the samples which were therefore physiologically more similar to each other at cellular level.

**Table 1:** Numbers of significantly enriched categories in GSEA

General biochemical and cellular categories from KEGG, GO, TRANSPATH and TRANSFAC						
	**Roots**			**Leaves**		

Comparisons	induced	repressed	∑	induced	repressed	∑

WT - Fe vs. + Fe	9 (4)	17 (11)	26 (15)	18 (11)	13 (4)	31 (15)
*nas4x-1 *- Fe vs. + Fe	5 (3)	3 (2)	8 (5)	10 (8)	2 (2)	12 (10)
+ Fe *nas4x-1 *vs. WT	16 (5)	5 (3)	21 (8)	3 (3)	2 (2)	5 (5)
- Fe *nas4x-1 *vs. WT	5 (2)	-	5 (2)	11 (6)	3 (2)	14 (8)

**MapMan categories**						

	**Roots**			**Leaves**		

Comparisons	induced	repressed	∑	induced	repressed	∑

WT - Fe vs. + Fe	5	1	6	1	-	1
*nas4x-1 *- Fe vs. + Fe	3	1	4	4	1	5
+ Fe *nas4x-1 *vs. WT	3	2	5	4	2	6
- Fe *nas4x-1 *vs. WT	4	-	4	3	4	7

The numbers were obtained by counting induced and repressed categories of Table S1 and Table S2. In brackets are the numbers of areas into which the corresponding enriched categories were grouped.

When comparing leaf samples the majority of categories were also affected between wild type + and - Fe (in total 31 categories belonging to 15 areas), while an intermediate number of categories was hit between *nas4x-1 *samples (in total twelve categories belonging to ten areas) and between *nas4x-1 *and wild type at - Fe (in total 14 categories belonging to eight areas) (Tables 1 and Additional file 3, Table S1). Few changes of categories were found between *nas4x-1 *and wild type leaves at + Fe (in total five categories belonging to five areas) (Tables 1 and Additional file 3, Table S1). These comparisons therefore suggest that wild type + and - Fe leaf samples were physiologically very different, whereas *nas4x-1 *leaf samples (+ or - Fe) and - Fe samples (*nas4x-1 *or wild type) were only partially physiologically distinct. Little physiological difference was detected between *nas4x-1 *and wild type leaves upon + Fe. Therefore, roots and leaves reacted with similar strength to + and - Fe. The *nas4x-1 *mutation had resulted in an approximation of the - Fe wild type situation in roots and of the + Fe wild type cell physiological situation in leaves.

Due to the diversity and little overlap of cellular categories hit in between the different comparisons we were not able to represent the results in Venn diagrams in any reasonable manner (not shown).

### GSEA of transcriptome data using specific plant physiology categories (MapMan)

The GeneTrail-predefined categories utilized in the previous paragraph reflected the physiological status at cellular level but did not appear sufficient for the investigation at whole organism level. To circumvent this obstacle, we performed GSEA with categories that had been developed for the plant-specific visualization tool MapMan [6]. MapMan categories could be incorporated into the GSEA tool of GeneTrail as individually defined categories. Contrary to the GeneTrail-predefined categories the genes of MapMan categories had been grouped according to physiological aspects and pathways relevant for plants.

**Figure 1:**
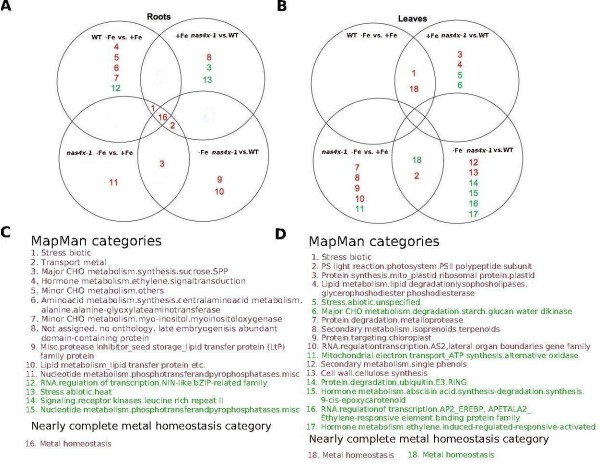
**Venn diagrams illustrating co-regulated functional categories (MapMan and metal homeostasis categories) in the eight pairwise comparisons of transcriptome data**. (A, B) Venn diagrams summarizing co-regulation data of enriched categories in pairwise comparisons of (A) root and (B) leaf transcriptome data. Each circle represents the pairwise comparison indicated. The numbers indicate the respective categories that were found enriched (see C, D). If categories were enriched in more than one comparison the respective number is found in the overlap region of the circles. (C, D) Designation of categories that were found enriched in (C) root comparisons and (D) leaf comparisons. Red coloured numbers indicate induced categories, green coloured numbers indicate repressed categories.

The number of MapMan categories affected in the eight meaningful comparisons was determined as in the previous paragraph (Tables 1 and Additional file 4, Table S2). We found that between one and seven MapMan categories (induced and repressed counted together) were hit in the eight comparisons (Tables 1 and Additional file 4, Table S2). The majority of MapMan categories affected was found when comparing wild type roots + and - Fe (six categories) and leaf *nas4x-1 *vs. wild type (six and seven categories for + and - Fe, respectively) (Table 1). Only one MapMan category was hit in the comparison of leaf + vs. - Fe, while all other comparisons gave intermediate numbers of MapMan categories hit (four to five) (Table 1). In total we identified 15 different MapMan categories in all comparisons of root samples and 17 different MapMan categories in all comparisons of leaf samples. The data were represented in Venn diagrams (Figure 1). This representation shows that among the 15 categories affected in root samples three MapMan categories were shared between at least two comparisons, namely biotic stress, metal transport and carbohydrate metabolism (Figure 1A, C). The biotic stress category was found induced in comparisons of - Fe vs. + Fe (in wild type and in *nas4x-1*) and in *nas4x-1 *vs. wild type at + Fe, indicating that biotic stress responses were generally induced by Fe deficiency. The metal transport category was induced in comparisons of *nas4x-1 *vs. wild type and between *nas4x-1 *- and + Fe, showing that metal transport processes were reoriented in *nas4x-1*. Finally, carbohydrate metabolism was induced in *nas4x-1 *- Fe vs. + Fe and vs. wild type - Fe suggesting that in *nas4x-1 *plants carbohydrate metabolism was altered in response to - Fe.

Among the 17 MapMan categories affected in leaf samples only two categories were hit in at least two comparisons as deduced from the Venn diagram (Figure 1B, D). The photosystem category was induced in leaves in the comparisons of *nas4x-1 *- Fe vs. + Fe and *nas4x-1 *vs. wild type at - Fe indicating that *nas4x-1 *leaves at - Fe experienced a remodeling of the photosynthetic apparatus. The MapMan category biotic stress was induced in wild type - Fe vs. + Fe and at + Fe in *nas4x-1 *vs. wild type indicating that - Fe conditions resulted in a need for stress defense.

This analysis indicated that the incorporation of plant-specific physiological categories into GSEA added possibilities for novel physiological interpretations at whole organism level that were not achieved by merely concentrating on cellular categories.

### GSEA of transcriptome data using an individually designed metal homeostasis category

Surprisingly, GSEA of MapMan categories did not reveal hits of the transport metal category in each of the eight meaningful comparisons. One possible explanation could be that metal transport was not affected in all comparisons. However, an alternative interpretation could be of technical nature that simply the transport metal MapMan category was not complete. Indeed, this MapMan category only contained 47 genes involved in uptake, transport and allocation of metal ions (further information at http://genetrail.bioinf.uni-sb.de/paper/ath/), whereas the list of published genes that were affected by altered metal distribution was larger. We intended therefore to test a large metal homeostasis category in GSEA. To obtain such a category, we collected a nearly complete set of genes assembled from published data of metal homeostasis genes and their homologous genes based on sequence similarities and created an individual, new functional category, that we named "metal homeostasis" (Additional file 5, Table S3; the gene list of this category is available as Additional file 6, Table S4). When performing GSEA this individually defined metal homeostasis category showed enrichment in all eight meaningful pairwise comparisons (Figure 1; results are available at http://genetrail.bioinf.uni-sb.de/paper/ath/). The category was found induced in all comparisons of root samples with - Fe vs. + Fe and *nas4x-1 *vs. wild type, as well as of leaf samples with wild type - Fe vs. + Fe and + Fe *nas4x-1 *vs. wild type (Figure 1). The category was repressed in leaf comparisons of *nas4x-1 *- Fe vs. + Fe and - Fe *nas4x-1 *vs. wild type (Figure 1).

Thus, changes in external Fe supply or in internal regulators of metal chelation and transport resulted in significant alterations of gene expression patterns of an entire category of genes representing the components for metal homeostasis.

### Over Representation Analysis (ORA) of 258 differentially expressed genes

Finally, we aimed at utilising GeneTrail to identify functional categories among selected significantly differentially expressed genes that could be revealed from our transcriptome data [19]. To identify a list of significantly differentially expressed genes we used the NIA array analysis software tool to analyze the eight meaningful pairwise comparisons. Root and leaf samples were considered separately from each other. The pairwise comparisons of expression values revealed a total number of 226 leaf-specific and 32 root-specific differentially expressed genes (Additional file 7, Table S5). These 258 genes showed a differential expression in at least one single pairwise comparison in the NIA Array analysis. With this data set we performed an Over Representation Analysis (ORA) to test whether among the 258 differentially expressed genes specific biological categories or pathways were affected. When an ORA was performed with the GeneTrail-predefined categories from KEGG, GO, TRANSPATH and TRANSFAC no category was enriched within the 258 selected genes compared to all the genes on the ATH1 gene chip. Upon ORA with MapMan categories seven MapMan categories were enriched (Table 2). Among the enriched categories were two metal specific categories, named "metalhandling, binding, chelation and storage" and "transport metal", two different oxidative stress categories, both named "redox.dismutases and catalases", a cell division, a GCN5-related N-acetyltransferase and a non-assigned category (Table 2). We also performed ORA with the metal homeostasis category that we have designed individually as described above. This category was found enriched as expected. Hence, we conclude from ORA analysis of the differentially expressed genes that metal homeostasis as a category was preferentially affected in our experimental conditions. In conclusion, ORA of pre-selected genes allowed to interpret transcriptome data in meaningful physiological contexts.

**Table 2:** Enriched MapMan categories testing the 258 NIA pre-selected genes compared to all the genes present on the ATH1 gene chip in the ORA

Enriched categories	Associated genes
metalhandling.binding, chelationandstorage	NAS3, ATCCS, ATFER4, ATFER3, CCH, ATFER1, NAS1, NAS2
redox.dismutasesandcatalases	ATCCS, CSD2, FSD1
redox.dismutasesandcatalases	WRKY60 WRKY46 WRKY47 WRKY53 WRKY48
transport.metal	NRAMP3, MTPA2, IRT2, ZIP5, HMA5, YSL1
cell.division	AT1G49910 AT1G69400 CDKB1;2 APC8 ATSMC3
misc.gcN5-related N-acetyltransferase	AT2G32020 AT2G32030 AT2G39030
notassigned.noontology	AT3G07720 AT5G52670 AT1G09450 CENP-C COR414-TM1ZW9 AT1G76260 ATNUDT6 ATEXO70H4 AT3G14100 ATNUDX13 AT4G36700

The table illustrates those genes among the 258 NIA preselected genes, which are associated with enriched categories.

## Discussion

Here, we mined comparative Arabidopsis transcriptome data and identified differentially regulated functional categories and pathways using the web-based tool GeneTrail, by performing Gene Set Enrichment Analysis (GSEA) of eight meaningful pairwise comparisons between leaf and root, *nas4x-1 *mutant versus wild type samples, in response to + vs. - Fe. From our data analysis we were able to determine differential numbers and types of enriched functional categories for the respective comparisons. Hence, we could characterize phenotypes at cell biological level, at whole-organism physiological level and with respect to metal homeostasis. 258 differentially expressed genes were identified from the eight meaningful pairwise comparisons. By Over-Representation Analysis (ORA) of these pre-selected genes we could determine that five plant physiological categories were overrepresented among them. The example we presented here can also be used as an outline that guides researchers through microarray analysis with the aim of identifying regulated functional categories of genes in plants. GeneTrail was found particularly useful for plant physiological analysis due to its feature that allowed incorporation of individually defined functional categories.

### Confirmation of molecular phenotypes by GSEA, and identification of differentially expressed categories

GSEA of general biochemical and cell biological categories demonstrated that roots and leaves of wild type plants had reacted with similar strength to - Fe. 26 and 31 categories in total were differentially regulated in wild type roots and leaves, respectively, between + and - Fe. This number of enriched categories was higher than that of any comparisons involving *nas4x-1 *samples. Multiple reasons may have accounted for differential regulation of these categories. Regulation of the category might indicate an adaptation to Fe deficiency stress such as for example defense responses. Alternatively, the lack of Fe as a cofactor for specific enzyme activities may have led to deregulated gene expression of these enzymes due to feedback control, such as for example oxidoreductase activity, nitrate and amino acid metabolism. The lowered photosynthetic activity at - Fe may also have caused extensive metabolic changes for production of anaerobic energy as represented for example by carbohydrate and energy categories.

The lowest numbers of differentially regulated categories were detected between roots - Fe, *nas4x-1 *vs. wild type, and leaves + Fe, *nas4x-1 *vs. wild type. We conclude from these numbers of regulated categories that + Fe *nas4x-1 *mutant root cells had approximated the cellular status present in - Fe wild type roots, while + Fe *nas4x-1 *mutant leaf cells had reacted closest to those of + Fe wild type cells. These findings correlated well with our previous analysis of the *nas4x-1 *mutant. Based on our previous investigation of Fe content, regulation of Fe deficiency genes, *YSL2 *transporter and ferritin genes we had proposed that the lack of nicotianamine had caused increased Fe deficiency responses in the root, but Fe deficiency and sufficiency responses in the leaves [14]. Although the comparison of the numbers of regulated cell biological categories was meaningful to us, the exact nature of these categories was not suitable for finding overlaps in regulatory patterns between different samples. Due to this lack of overlaps we were not able to represent the results in Venn diagrams. One possible explanation for this puzzling finding could be that the cell biological categories contained mostly rather few genes so that the diversity of categories was high. Perhaps if the high number of general categories derived from KEGG, GO, TRANSFAC and TRANSPATH was reassembled into areas each comprising several of the categories more overlap in regulatory patterns may become apparent, e.g. through assembly of individual pyrimidine, purine and nucleoside metabolism into a large nucleoside/nucleotide metabolism category, or of individual leucine, tyrosine, etc. categories into a large N metabolism category.

Interestingly, the above conclusion about the cell physiological status of mutant and wild type situations was not possible when analyzing MapMan plant physiological categories. In those cases, a low number of differentially expressed categories was found for the comparison of wild type, + vs. - Fe, whereas the highest number was revealed in the comparison of - Fe, *nas4x-1 *vs. wild type. A reason could be that the enriched plant physiological MapMan categories had represented adaptations to + or - Fe, mutant or wild type at whole organ level rather than at cellular level, such as for example stress responses. On the other hand, the MapMan categories comprised plant-specific categories like plant hormone metabolism and regulation which could be made responsible for conferring adaptations at cellular level so that cellular differences became more or less apparent.

GSEA with a nearly complete metal homeostasis category showed that in all meaningful pairwise comparisons, between + and - Fe, wild type and *nas4x-1 *samples, metal homeostasis was found affected. The metal homeostasis category contained many genes involved in metal transport or metal regulation assembled from studies reporting mainly their up-regulation in response to - Fe. From the observation that this category was found induced in wild type - vs. + Fe in roots and in leaves we can deduce that indeed the metal homeostasis category was an indicator for Fe deficiency responses. In all root comparisons of *nas4x-1 *vs. wild type and of - Fe vs. + Fe this category was induced and hence the *nas4x-1 *mutant status of roots can be considered Fe-deficient, in agreement with the above findings on cell biological categories and the previous findings reported [14]. On the other hand, we have previously determined that *nas4x-1 *leaf cells showed partially signs of Fe deficiency and partially of Fe sufficiency. This was reflected by the observation that in the comparisons of leaf samples the metal homeostasis category was found induced and repressed, respectively.

Only from GSEA results of MapMan and the metal homeostasis categories we were able to construct meaningful Venn diagrams that revealed overlaps in regulatory patterns between the different samples. In roots and partially in leaves (under - Fe vs. + Fe) and at + Fe (*nas4x-1 *vs. wild type) we found induction of the biotic stress category, indicative of an adaptation to avoid pathogen infection under - Fe. Carbohydrate metabolism was also affected in multiple pairwise comparisons indicative of altered sugar utilization due to reduced photosynthesis at - Fe. In leaves, photosystem regulation was apparent as major regulated category. Hence, the metal homeostasis, biotic stress, root carbohydrate and leaf photosystem categories were the main targets for regulated changes in response to - Fe and *nas4x-1*.

### Identification of major regulated categories among differentially expressed genes using a combination of ORA and GSEA

The above discussed GSEA results might have masked regulated categories if they contained few differentially regulated genes but a high number of unregulated genes. To circumvent this potential obstacle we identified from our transcriptome data all genes that were differentially expressed in any of the meaningful pairwise comparisons and performed Over-Representation Analysis (ORA). None of the general cell biological categories was over-represented among these 258 genes. An explanation for this finding could be again that the categories from KEGG, GO, TRANSFAC and TRANSPATH were too low in size, unspecific and diverse for statistical analysis. On the other hand, ORA with MapMan categories identified several meaningful functional pathways differentially regulated in response to Fe supply and *nas4x-1*. In addition to metal homeostasis categories, this analysis revealed redox dismutase and catalase categories, a cell division and a GCN5-related N-acetyltransferase category. The reappearance of the metal homeostasis categories not only in GSEA but also in ORA shows again how significantly this pathway was affected in the transcriptome comparisons. As discussed above, an influence of - Fe and of *nas4x-1 *on metal homeostasis was expected from previous analysis and represented here a positive control for proper functioning of the GeneTrail tool. Redox dismutase and catalase genes were differentially regulated presumably because these enzymes often use Fe as cofactor. Low enzyme activity at - Fe may have resulted in differential expression as the result of a feedback control. Alternatively, upon - Fe new enzyme isoforms with different metal requirements might have been produced. It is also reasonable to argue that decreased Fe toxicity upon - Fe might have been the cause for the differential regulation of these genes. The differentially regulated cell division category may have reflected an adaptation of root growth behaviour. Finally, the GCN5-related N-acetyltransferase category represented specifically genes involved in histone acetylation, a process associated generally with gene activation. This study and others have shown that - Fe conditions caused an up-regulation of genes and proteins that was more important than a down-regulation [22-24]. It is therefore plausible that genes and enzymes involved in histone acetylation were activated to render more chromosomal areas accessible to the transcription machineries.

## Conclusion

Analysis of differentially regulated functional categories confirmed that the *nas4x-1 *mutant is defective in metal homeostasis. The mutant was found to show Fe deficiency signs in roots and signs of Fe deficiency and Fe sufficiency in leaves. Biotic stress, root carbohydrate, leaf photosystem and specific cell biological categories were also discovered as main targets for regulated changes in response to - Fe and *nas4x-1*. 258 genes differentially expressed in response to - Fe and *nas4x-1 *were identified. Among these genes, five functional categories were enriched including metal transport and metal binding, redox regulation, cell division and histone acetylation. GeneTrail is therefore generally highly suitable to reveal functional categories among comparative transcriptome data in Arabidopsis. We could use the quantitative and qualitative aspects provided by GSEA to interpret molecular-physiological phenotypes. A combination of the GeneTrail analysis methods, GSEA and ORA, together with other analysis tools, like the NIA array tool, was successfully applied for data mining. The main strength of GeneTrail was that it offered answers to individual biological questions with its feature of incorporation of individually defined categories (such as MapMan and metal homeostasis). Hence, GeneTrail can be applied to analyze novel physiological treatments or unknown mutations to identify functional pathways that are affected.

## Web links

### GeneTrail

http://genetrail.bioinf.uni-sb.de/

### NIA Array Analysis

http://lgsun.grc.nia.nih.gov/ANOVA/

### Web-site containing links to GSEA and ORA results

http://genetrail.bioinf.uni-sb.de/paper/ath/

## Authors' contributions

MS drafted the manuscript, established the experimental design and conducted the experimental work, performed plant growth, sample preparation and data analysis. KP performed the microarray work and revised the manuscript critically. AK performed pre-processing and statistical analysis of the microarray data. CB conducted adaptations of the GeneTrail software for the use of *Arabidopsis thaliana*. CB and AK supported the application of GeneTrail and revised the manuscript critically. HPL supervised the computational work on the GeneTrail software. PB conceived, designed and supervised the experimental design and participated in drafting the manuscript. All authors have read and approved the final manuscript.

## Abbreviations

GO: gene ontology; GSEA: Gene set enrichment analysis; KEGG: Kyoto enzyclopedia of genes and genomes; NA: nicotianamine; ORA: Over-representation analysis

## Supplementary Material

Additional file 1 **Figure S1: Overview of the experimental set-up**. (A) Scheme showing three biological repetitions (R1, R2, R3) harvested in three consecutive weeks for the microarray experiment. (B) Images of *nas4x-1 *and wild type plants grown for four weeks under Fe supply (10 μM Fe) and one week under Fe supply or Fe deficiency (0 Fe) conditions, respectively. (C) Work flow of transcriptome and bioinformatic analysis. (D) Eight meaningful comparisons for root and leaf samples. Click here for file

Additional file 2 **Figure S2: Types of running sum statistics when applying a Gene Set Enrichment Analysis**. (A) Mountain-like graph; in this example the enriched category "iron ion binding" illustrates a mountain-like graph for top-ranked genes in the comparison of wild type leaves + Fe vs. - Fe, indicating that genes of this category were mostly induced at + Fe. (B) Valley-like graph; in this example the enriched category "Golgi vesicle transport" illustrates a valley-like graph for bottom-ranked genes in the comparison of wild type roots + Fe vs. - Fe, indicating that genes of this category were mostly repressed under + Fe. Click here for file

Additional file 3 **Table S1: Selection of significantly enriched categories in the GSEA using GeneTrail-predefined GO, KEGG, TRANSPATH and TRANSFAC categories**. Click here for file

Additional file 4 **Table S2: Selection of significantly enriched categories in the GSEA using MapMan categories**. Click here for file

Additional file 5 **Table S3: Annotated gene list of the self-defined category "metal homeostasis"**. Click here for file

Additional file 6 **Table S4: Gene list of the self-defined category metal homeostasis.txt**. Click here for file

Additional file 7 **Table S5: Gene list of 258 NIA selected genes.txt**. Click here for file
