# Supplementary material for: Transcriptome analysis by GeneTrail revealed regulation of functional categories in response to alterations of iron homeostasis in Arabidopsis thaliana
Source: BMC Plant Biol. 2011 May 18;11:87. doi: 10.1186/1471-2229-11-87 (PMC3114716; doi:10.1186/1471-2229-11-87)
Supplement: Additional file 1 — Figure S1: Overview of the experimental set-up. (A) Scheme showing three biological repetitions (R1, R2, R3) harvested in three consecutive weeks for the microarray experiment. (B) Images of nas4x-1 and wild type plants grown for four weeks under Fe supply (10 μM Fe) and one week under Fe supply or Fe deficiency (0 Fe) conditions, respectively. (C) Work flow of transcriptome and bioinformatic analysis. (D) Eight meaningful comparisons for root and leaf samples. [file 1471-2229-11-87-S1.PDF]

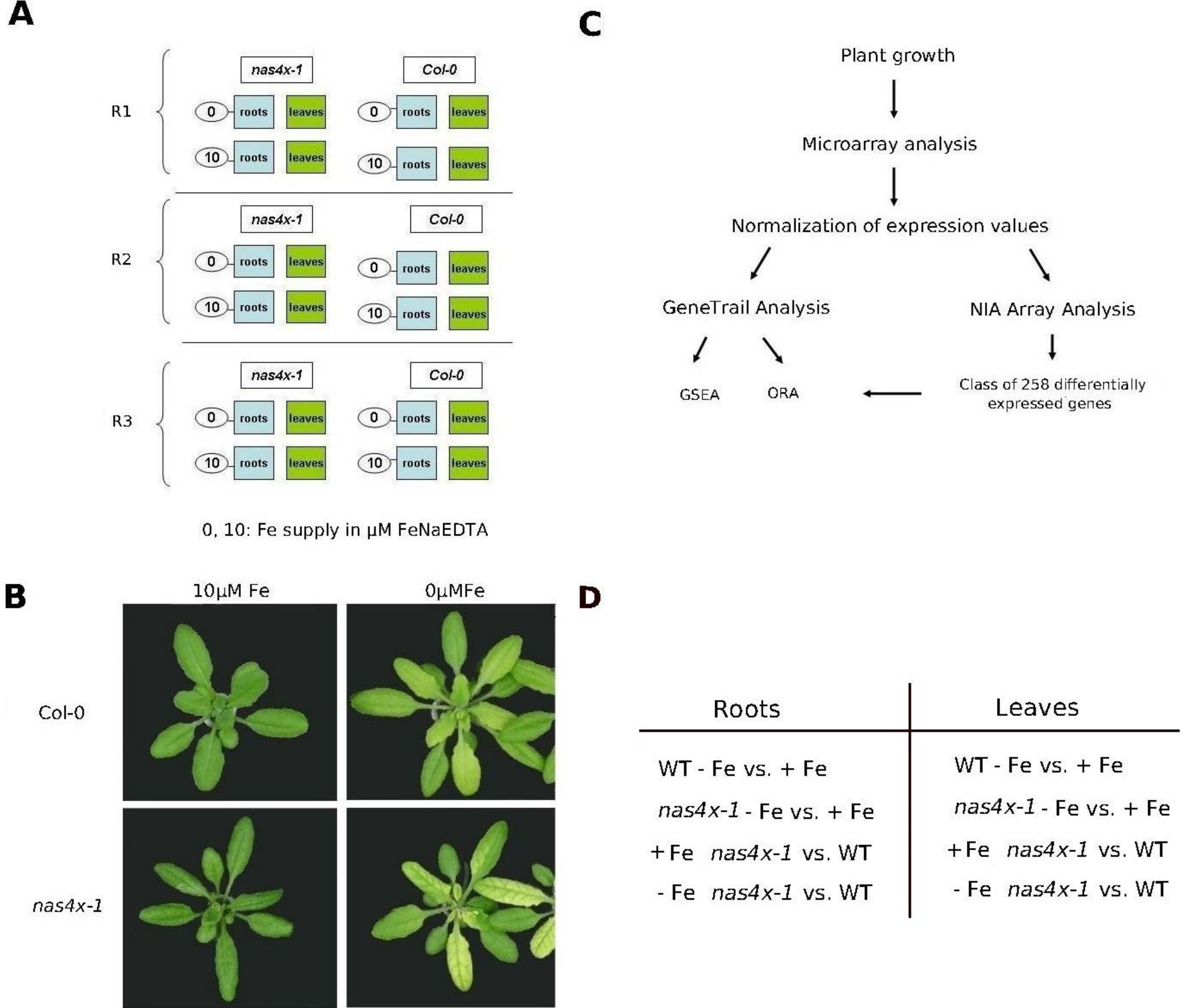
A
R1
R2
R3
nas4x-1
Col-0
0
10
roots
leaves
0, 10: Fe supply in µM FeNaEDTA
B
10µM Fe
0µMFe
Col-0
nas4x-1
C
Plant growth
Microarray analysis
Normalization of expression values
GeneTrail Analysis
NIA Array Analysis
GSEA
ORA
Class of 258 differentially expressed genes
D
Roots
Leaves
WT - Fe vs. + Fe
nas4x-1 - Fe vs. + Fe
+Fe nas4x-1 vs. WT
- Fe nas4x-1 vs. WT
