# Supplementary figures and images for: Transcriptome analysis by GeneTrail revealed regulation of functional categories in response to alterations of iron homeostasis in Arabidopsis thaliana
Source: BMC Plant Biol. 2011 May 18;11:87. doi: 10.1186/1471-2229-11-87 (PMC3114716; doi:10.1186/1471-2229-11-87)

**A**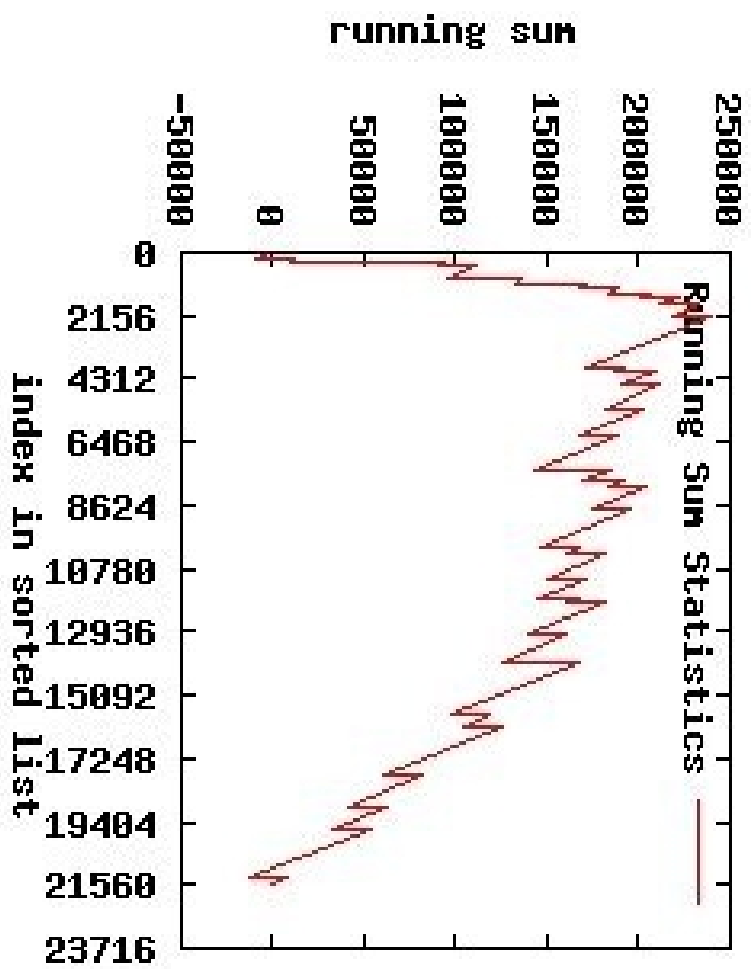**B**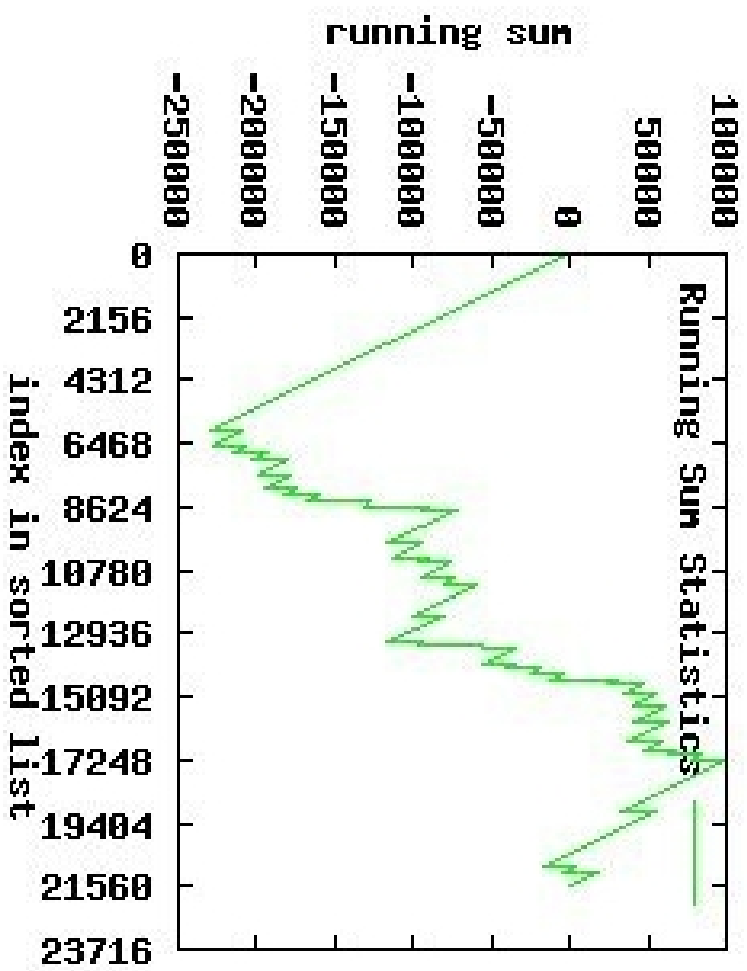

Supplement: Additional file 2 — Figure S2: Types of running sum statistics when applying a Gene Set Enrichment Analysis. (A) Mountain-like graph; in this example the enriched category "iron ion binding" illustrates a mountain-like graph for top-ranked genes in the comparison of wild type leaves + Fe vs. - Fe, indicating that genes of this category were mostly induced at + Fe. (B) Valley-like graph; in this example the enriched category "Golgi vesicle transport" illustrates a valley-like graph for bottom-ranked genes in the comparison of wild type roots + Fe vs. - Fe, indicating that genes of this category were mostly repressed under + Fe. [file 1471-2229-11-87-S2.PDF]
