# Supplementary material for: Transcriptome analysis by GeneTrail revealed regulation of functional categories in response to alterations of iron homeostasis in Arabidopsis thaliana
Source: BMC Plant Biol. 2011 May 18;11:87. doi: 10.1186/1471-2229-11-87 (PMC3114716; doi:10.1186/1471-2229-11-87)
Supplement: Additional file 3 — Table S1: Selection of significantly enriched categories in the GSEA using GeneTrail-predefined GO, KEGG, TRANSPATH and TRANSFAC categories. [file 1471-2229-11-87-S3.DOC]

| **Table S1: Selection of significantly enriched categories in the GSEA using GeneTrail-predefined GO, KEGG, TRANSPATH and TRANSFAC categories** | |
| --- | --- |
| **WT – Fe vs. + Fe, roots** | |
| **Induced** | **p-value** |
| energy reserve metabolic process | 0.011926 |
| oxidoreductase activity | 0.0239756 |
| carbohydrate phosphatase activity | 0.0249961 |
| hypersensitive response | 0.0319398 |
| leucine metabolic process | 0.0331979 |
| tyrosine transaminase activity | 0.0377122 |
| response to nitrate | 0.0382981 |
| regulation of cellular defense response | 0.0462234 |
| leucine catabolic process | 0.0492772 |
| **Repressed** | **p-value** |
| [dolichol metabolic process](http://www.godatabase.org/cgi-bin/amigo/go.cgi?view=details&search_constraint=terms&depth=0&query=GO:0019348) | 0.00124733 |
| cold acclimation | 0.0015228 |
| [polyprenol biosynthetic process](http://www.godatabase.org/cgi-bin/amigo/go.cgi?view=details&search_constraint=terms&depth=0&query=GO:0016094) | 0.00247948 |
| [polyprenol metabolic process](http://www.godatabase.org/cgi-bin/amigo/go.cgi?view=details&search_constraint=terms&depth=0&query=GO:0016093) | 0.00247948 |
| [prenol biosynthetic process](http://www.godatabase.org/cgi-bin/amigo/go.cgi?view=details&search_constraint=terms&depth=0&query=GO:0016091) | 0.00247948 |
| [prenol metabolic process](http://www.godatabase.org/cgi-bin/amigo/go.cgi?view=details&search_constraint=terms&depth=0&query=GO:0016090) | 0.00247948 |
| [chloroplast stromal thylakoid](http://www.godatabase.org/cgi-bin/amigo/go.cgi?view=details&search_constraint=terms&depth=0&query=GO:0009533) | 0.00422552 |
| [flavonol metabolic process](http://www.godatabase.org/cgi-bin/amigo/go.cgi?view=details&search_constraint=terms&depth=0&query=GO:0051554) | 0.0132765 |
| [flavonol biosynthetic process](http://www.godatabase.org/cgi-bin/amigo/go.cgi?view=details&search_constraint=terms&depth=0&query=GO:0051555) | 0.0352447 |
| [nucleoside diphosphate kinase activity](http://www.godatabase.org/cgi-bin/amigo/go.cgi?view=details&search_constraint=terms&depth=0&query=GO:0004550) | 0.0246695 |
| [COPI vesicle coat](http://www.godatabase.org/cgi-bin/amigo/go.cgi?view=details&search_constraint=terms&depth=0&query=GO:0030126) | 0.0249961 |
| [COPI-coated vesicle](http://www.godatabase.org/cgi-bin/amigo/go.cgi?view=details&search_constraint=terms&depth=0&query=GO:0030137) | 0.0249961 |
| [COPI coated vesicle membrane](http://www.godatabase.org/cgi-bin/amigo/go.cgi?view=details&search_constraint=terms&depth=0&query=GO:0030663) | 0.0249961 |
| [cellulase activity](http://www.godatabase.org/cgi-bin/amigo/go.cgi?view=details&search_constraint=terms&depth=0&query=GO:0008810) | 0.0271984 |
| [fatty acid (omega-1)-hydroxylase activity](http://www.godatabase.org/cgi-bin/amigo/go.cgi?view=details&search_constraint=terms&depth=0&query=GO:0008393) | 0.0292809 |
| [phototropism](http://www.godatabase.org/cgi-bin/amigo/go.cgi?view=details&search_constraint=terms&depth=0&query=GO:0009638) | 0.0447523 |
| [DNA polymerase complex](http://www.godatabase.org/cgi-bin/amigo/go.cgi?view=details&search_constraint=terms&depth=0&query=GO:0042575) | 0.0458627 |
| ***nas4x-1* – Fe vs. +Fe, roots** | |
| **Induced** | **p-value** |
| iron ion transmembrane transporter activity | 0.000668928 |
| endopeptidase inhibitor activity | 0.00604512 |
| protease inhibitor activity | 0.0121772 |
| secondary metabolic process | 0.0141253 |
| serine-type endopeptidase inhibitor activityacyl-CoA oxidase activity | 0.0212183 |
| **Repressed** | **p-value** |
| auxin polar transport | 0.00670236 |
| hormone transport | 0.00670236 |
| tubulin complex | 0.0154222 |
| **+ Fe *nas4x-1* vs. WT, roots** | |
| **Induced** | **p-value** |
| pyrimidine ribonucleoside metabolic process | 0.0109404 |
| [nutrient reservoir activity](http://www.godatabase.org/cgi-bin/amigo/go.cgi?view=details&search_constraint=terms&depth=0&query=GO:0045735) | 0.0128178 |
| pyrimidine nucleoside metabolic process | 0.0359885 |
| cytidine catabolic process | 0.0359885 |
| nucleoside catabolic process | 0.0359885 |
| cytidine deamination | 0.0359885 |
| ribonucleoside catabolic process | 0.0359885 |
| cytidine metabolic process | 0.0359885 |
| pyrimidine ribonucleoside catabolic process | 0.0109404 |
| pyrimidine nucleoside catabolic process | 0.0359885 |
| Response to metal ion | 0.00104659 |
| glycosinolate biosynthetic process | 0.00732211 |
| chitinase activity | 0.0013411 |
| [zinc ion transmembrane transporter activity](http://www.godatabase.org/cgi-bin/amigo/go.cgi?view=details&search_constraint=terms&depth=0&query=GO:0005385) | 0.00841823 |
| zinc ion transport | 0.0265626 |
| [isocitrate dehydrogenase (NAD+) activity](http://www.godatabase.org/cgi-bin/amigo/go.cgi?view=details&search_constraint=terms&depth=0&query=GO:0004449) | 0.0497346 |
| **Repressed** | **p-value** |
| sucrose-phosphate synthase activity | 0.0144042 |
| fatty acid (omega-1)-hydroxylase activity | 0.00266194 |
| [regulation of protein complex assembly](http://www.godatabase.org/cgi-bin/amigo/go.cgi?view=details&search_constraint=terms&depth=0&query=GO:0043254) | 0.0122457 |
| [regulation of cellular component biogenesis](http://www.godatabase.org/cgi-bin/amigo/go.cgi?view=details&search_constraint=terms&depth=0&query=GO:0044087) | 0.0122457 |
| [regulation of protein polymerization](http://www.godatabase.org/cgi-bin/amigo/go.cgi?view=details&search_constraint=terms&depth=0&query=GO:0032271) | 0.0270636 |
| **-Fe *nas4x-1* vs. WT, roots** |  |
| **Induced** | **p-value** |
| iron ion transmembrane transporter activity | 0.00434023 |
| hydrogen ion transporting ATP synthase activity | 0.0187568 |
| transition metal ion transmembrane transporter activity | 0.0293041 |
| hydrogen ion transporting ATPase activity | 0.0491874 |
| No repressed categories |  |
| **WT – Fe vs. + Fe, leaves** |  |
| **Induced** | **p-value** |
| [endoplasmic reticulum lumen](http://www.godatabase.org/cgi-bin/amigo/go.cgi?view=details&search_constraint=terms&depth=0&query=GO:0005788) | 0.00386982 |
| [histone phosphorylation](http://www.godatabase.org/cgi-bin/amigo/go.cgi?view=details&search_constraint=terms&depth=0&query=GO:0016572) | 0.00400061 |
| [chitinase activity](http://www.godatabase.org/cgi-bin/amigo/go.cgi?view=details&search_constraint=terms&depth=0&query=GO:0004568) | 0.00497135 |
| [spindle organization and biogenesis](http://www.godatabase.org/cgi-bin/amigo/go.cgi?view=details&search_constraint=terms&depth=0&query=GO:0007051) | 0.00513365 |
| [spindle assembly](http://www.godatabase.org/cgi-bin/amigo/go.cgi?view=details&search_constraint=terms&depth=0&query=GO:0051225) | 0.00513365 |
| [nitrate metabolic process](http://www.godatabase.org/cgi-bin/amigo/go.cgi?view=details&search_constraint=terms&depth=0&query=GO:0042126) | 0.00662199 |
| [nitrate assimilation](http://www.godatabase.org/cgi-bin/amigo/go.cgi?view=details&search_constraint=terms&depth=0&query=GO:0042128) | 0.00662199 |
| [DNA methylation on cytosine](http://www.godatabase.org/cgi-bin/amigo/go.cgi?view=details&search_constraint=terms&depth=0&query=GO:0032776) | 0.00859903 |
| [maintenance of DNA methylation](http://www.godatabase.org/cgi-bin/amigo/go.cgi?view=details&search_constraint=terms&depth=0&query=GO:0010216) | 0.0178867 |
| [multidrug transporter activity](http://www.godatabase.org/cgi-bin/amigo/go.cgi?view=details&search_constraint=terms&depth=0&query=GO:0015239) | 0.0196313 |
| [histone kinase activity](http://www.godatabase.org/cgi-bin/amigo/go.cgi?view=details&search_constraint=terms&depth=0&query=GO:0035173) | 0.0230892 |
| [gibberellin 3-beta-dioxygenase activity](http://www.godatabase.org/cgi-bin/amigo/go.cgi?view=details&search_constraint=terms&depth=0&query=GO:0016707) | 0.0230892 |
| Nutrient reservoir activity | 0.0273294 |
| [calcium:hydrogen antiporter activity](http://www.godatabase.org/cgi-bin/amigo/go.cgi?view=details&search_constraint=terms&depth=0&query=GO:0015369) | 0.0377138 |
| [metal ion:hydrogen antiporter activity](http://www.godatabase.org/cgi-bin/amigo/go.cgi?view=details&search_constraint=terms&depth=0&query=GO:0051139) | 0.0377138 |
| [systemic acquired resistance](http://www.godatabase.org/cgi-bin/amigo/go.cgi?view=details&search_constraint=terms&depth=0&query=GO:0009627) | 0.0377138 |
| [proteasome regulatory particle, lid subcomplex (sensu Eukaryota)](http://www.godatabase.org/cgi-bin/amigo/go.cgi?view=details&search_constraint=terms&depth=0&query=GO:0008541) | 0.0420145 |
| Golgi vesicle transport | 0.0373162 |
| **Repressed** | **p-value** |
| [glucan catabolic process](http://www.godatabase.org/cgi-bin/amigo/go.cgi?view=details&search_constraint=terms&depth=0&query=GO:0009251) | 0.00335301 |
| [starch catabolic process](http://www.godatabase.org/cgi-bin/amigo/go.cgi?view=details&search_constraint=terms&depth=0&query=GO:0005983) | 0.00335301 |
| [adenylate dimethylallyltransferase activity](http://www.godatabase.org/cgi-bin/amigo/go.cgi?view=details&search_constraint=terms&depth=0&query=GO:0009824) | 0.0105776 |
| [GTP biosynthetic process](http://www.godatabase.org/cgi-bin/amigo/go.cgi?view=details&search_constraint=terms&depth=0&query=GO:0006183) | 0.0230892 |
| [UTP biosynthetic process](http://www.godatabase.org/cgi-bin/amigo/go.cgi?view=details&search_constraint=terms&depth=0&query=GO:0006228) | 0.0230892 |
| [CTP biosynthetic process](http://www.godatabase.org/cgi-bin/amigo/go.cgi?view=details&search_constraint=terms&depth=0&query=GO:0006241) | 0.0230892 |
| [UTP metabolic process](http://www.godatabase.org/cgi-bin/amigo/go.cgi?view=details&search_constraint=terms&depth=0&query=GO:0046051) | 0.0230892 |
| [pyrimidine ribonucleoside triphosphate biosynthetic process](http://www.godatabase.org/cgi-bin/amigo/go.cgi?view=details&search_constraint=terms&depth=0&query=GO:0009209) | 0.0230892 |
| [GTP metabolic process](http://www.godatabase.org/cgi-bin/amigo/go.cgi?view=details&search_constraint=terms&depth=0&query=GO:0046039) | 0.0230892 |
| [CTP metabolic process](http://www.godatabase.org/cgi-bin/amigo/go.cgi?view=details&search_constraint=terms&depth=0&query=GO:0046036) | 0.0230892 |
| [sugar mediated signaling](http://www.godatabase.org/cgi-bin/amigo/go.cgi?view=details&search_constraint=terms&depth=0&query=GO:0010182) | 0.0231665 |
| [iron ion binding](http://www.godatabase.org/cgi-bin/amigo/go.cgi?view=details&search_constraint=terms&depth=0&query=GO:0005506) | 0.0289028 |
| [pathogenesis](http://www.godatabase.org/cgi-bin/amigo/go.cgi?view=details&search_constraint=terms&depth=0&query=GO:0009405) | 0.0483069 |
| ***nas4x-1* – Fe vs. + Fe, leaves** | |
| **Induced** | **p-value** |
| large ribosomal subunit | 0.00337326 |
| transcription factor import into nucleus | 0.00484658 |
| magnesium ion binding | 0.0068359 |
| metallopeptidase activity | 0.00737924 |
| plant-type secondary cell wall biogenesis | 0.0121333 |
| toxin receptor binding | 0.0234517 |
| microsporogenesis | 0.0289648 |
| ncRNA processing | 0.0351721 |
| histone phosphorylation | 0.0390928 |
| oxygen evolving complex | 0.042861 |
| **Repressed** | **p-value** |
| inorganic phosphate transmembrane transporter activity | 0.00196892 |
| alternative oxidase activity | 0.00209068 |
| **+ Fe *nas4x-1* vs. WT, leaves** | |
| **Induced** | **p-value** |
| 1-aminocyclopropane-1-carboxylate synthase activity | 0.0067324 |
| response to singlet oxygen | 0.0201692 |
| [endoplasmic reticulum lumen](http://www.godatabase.org/cgi-bin/amigo/go.cgi?view=details&search_constraint=terms&depth=0&query=GO:0005788) | 0.00156318 |
| **Repressed** | **p-value** |
| phosphotransferase activity, paired acceptors | 0.04908 |
| autophagy | 0.0247019 |
| **- Fe *nas4x-1* vs. WT, leaves** |  |
| **Induced** | **p-value** |
| synapsis | 0.000963428 |
| microtubule organizing center | 0.00158795 |
| histone phosphorylation | 0.0121133 |
| spindle organization and biogenesis | 0.0150223 |
| maintenance of DNA methylation | 0.0227776 |
| regulation of abscisic acid mediated signaling | 0.0230474 |
| gibberellin 2-beta-dioxygenase activity | 0.0242852 |
| vacuolar protein processing | 0.0272157 |
| nitrate assimilation | 0.0307466 |
| carotenoid dioxygenase activity | 0.0359393 |
| histone kinase activity | 0.0466719 |
| **Repressed** | **p-value** |
| DNA replication initiation | 0.0353621 |
| cation:chloride symporter activity | 0.0392881 |
| replication fork | 0.0466718 |
