# Supplementary material for: Transcriptome analysis by GeneTrail revealed regulation of functional categories in response to alterations of iron homeostasis in Arabidopsis thaliana
Source: BMC Plant Biol. 2011 May 18;11:87. doi: 10.1186/1471-2229-11-87 (PMC3114716; doi:10.1186/1471-2229-11-87)
Supplement: Additional file 4 — Table S2: Selection of significantly enriched categories in the GSEA using MapMan categories. [file 1471-2229-11-87-S4.DOC]

| **Table S2: Selection of significantly enriched categories in the GSEA using MapMan categories** | |
| --- | --- |
| **WT – Fe vs. + Fe, roots** | |
| **Induced** | **p-value** |
| stress.biotic | 0 |
| hormonemetabolism.ethylene.signaltransduction | 0.00185945 |
| minorCHOmetabolism.others | 0.0142818 |
| aminoacidmetabolism.synthesis. centralaminoacidmetabolism. alanine.alanine-glyoxylateaminotransferase | 0.0379827 |
| minorCHOmetabolism.myo-inositol.myoinositoloxygenases | 0.0379827 |
| **Repressed** | **p-value** |
| RNA.regulationoftranscription.NIN-likebZIP-relatedfamily | 0.0396559 |
| ***nas4x-1* – Fe vs. + Fe, roots** | |
| **Induced** | **p-value** |
| stress.biotic | 0 |
| transport.metal | 0.0294365 |
| major CHOmetabolism.synthesis.sucrose.SPP | 0.0377283 |
| **Repressed** | **p-value** |
| nucleotidemetabolism.phosphotransferandpyrophosphatases.misc | 0.00951905 |
| **+ Fe *nas4x-1* vs. WT, roots** |  |
| **Induced** | **p-value** |
| stress.biotic | 0.0165565 |
| transport metal | 0.00370836 |
| notassigned.noontology.lateembryogenesisabundantdomain-containingprotein | 0.0415224 |
| **Repressed** | **p-value** |
| majorCHOmetabolism.synthesis.sucrose.SPS | 0.00370836 |
| signalling.receptorkinases.leucinerichrepeatII | 0.0421146 |
| **- Fe *nas4x-1* vs. WT, roots** |  |
| **Induced** | **p-value** |
| transport.metal | 0.0394575 |
| misc.protease inhibitor_seed storage_lipid transfer protein (LtP) family protein | 0.0394575 |
| major CHO metabolism.synthesis.sucrose.SPP | 0.0486378 |
| lipid metabolism.lipid transfer proteins etc | 0.0486378 |
| **No category repressed** | |
| **WT – Fe vs. + Fe, leaves** |  |
| **Induced** | **p-value** |
| stress.biotic | 0 |
| **No category repressed** | |
| ***nas4x-1* – Fe vs. + Fe, leaves** |  |
| **Induced** | **p-value** |
| secondarymetabolism.isoprenoids.terpenoids | 0.020535 |
| protein.tarGetinG.chloroplast | 0.0258094 |
| RNA.reGulationoftranscription.AS2,LateralOrGanBoundariesGeneFamily | 0.0258094 |
| PS.liGhtreaction.photosystemII.PSIIpolypeptidesubunits | 0.0480656 |
| **Repressed** | **p-value** |
| mitochondrialelectrontransport_AtPsynthesis.alternativeoxidase | 0.0159923 |
| **+ Fe *nas4x-1* vs. WT, leaves** |  |
| **Induced** | **p-value** |
| stress.biotic | 0.0188724 |
| protein.synthesis.mito_plastid ribosomal protein.plastid | 0 |
| lipid metabolism.lipid degradation.lysophospholipases.glycerophosphodiester phosphodiesterase | 0.0334647 |
| tetrapyrrole synthesis.magnesium chelatase |  |
| **Repressed** | **p-value** |
| stress.abiotic.unspecified | 0.0334647 |
| major CHO metabolism.degradation.starch.glucan water dikinase | 0.0346834 |
| **- Fe *nas4x-1* vs. WT, leaves** |  |
| **Induced** | **p-value** |
| PS.lightreaction.photosystem II.PSII polypeptide subunits | 0.00055485 |
| secondary metabolism.simple phenols | 0.011214 |
| cell wall.cellulose synthesis | 0.0366933 |
| **Repressed** | **p-value** |
| protein.degradation.ubiquitin.E3.RING | 0 |
| hormone metabolism.abscisic acid.synthesis-degradation.synthesis.9-cis-epoxycarotenoid dioxygenase | 0.0165078 |
| RNA.regulation of transcription.AP2_EREBP, APEtALA2_Ethylene-responsive element binding protein family | 0.0200068 |
| hormone metabolism.ethylene.induced-regulated-responsive-activated | 0.0281475 |
