# Supplementary material for: Transcriptome analysis by GeneTrail revealed regulation of functional categories in response to alterations of iron homeostasis in Arabidopsis thaliana
Source: BMC Plant Biol. 2011 May 18;11:87. doi: 10.1186/1471-2229-11-87 (PMC3114716; doi:10.1186/1471-2229-11-87)
Supplement: Additional file 5 — Table S3: Annotated gene list of the self-defined category "metal homeostasis". [file 1471-2229-11-87-S5.DOC]

| **Table S3: Annotated gene list of the self-defined category “metal homeostasis“** | | |
| --- | --- | --- |
| **AGI ID** | **Affy ID** | **Annotation** |
| AT5G24380 | 249759_at | ATYSL2__YSL2 (YELLOW STRIPE LIKE 2); oligopeptide transporter |
| AT5G53550 | 248276_at | ATYSL3__YSL3 (YELLOW STRIPE LIKE 3); oligopeptide transporter |
| AT5G41000 | 249334_at | YSL4 (YELLOW STRIPE LIKE 4); oligopeptide transporter |
| AT3G17650 | 258353_s_at | PDE321__YSL5 (YELLOW STRIPE LIKE 5); oligopeptide transporter |
| AT3G27020 | 257789_at | YSL6 (YELLOW STRIPE LIKE 6); oligopeptide transporter |
| AT1G65730 | 262925_at | YSL7 (YELLOW STRIPE LIKE 7); oligopeptide transporter |
| AT1G48370 | 262248_at | YSL8 (YELLOW STRIPE LIKE 8); oligopeptide transporter |
| AT4G19690 | 254550_at | IRT1 (iron-regulated transporter 1); cadmium ion transmembrane transporter/ copper uptake transmembrane transporter/ iron ion transmembrane transporter/ manganese ion transmembrane transporter/ zinc ion transmembrane transporter |
| AT4G19680 | 254534_at | IRT2; iron ion transmembrane transporter/ zinc ion transmembrane transporter |
| AT3G12750 | 257715_at | ZIP1 (ZINC TRANSPORTER 1 PRECURSOR); zinc ion transmembrane transporter |
| AT5G59520 | 247678_at | ZIP2; copper ion transmembrane transporter/ transferase, transferring glycosyl groups / zinc ion transmembrane transporter |
| AT2G32270 | 266336_at | ZIP3; zinc ion transmembrane transporter |
| AT1G10970 | 260462_at | ATZIP4__ZIP4 (ZINC TRANSPORTER 4 PRECURSOR); cation transmembrane transporter/ copper ion transmembrane transporter |
| AT1G05300 | 264574_at | ZIP5; cation transmembrane transporter/ metal ion transmembrane transporter |
| AT2G30080 | 267304_at | ATZIP6__ZIP6; cation transmembrane transporter/ metal ion transmembrane transporter |
| AT2G04032 | 263480_at | ZIP7 (ZINC TRANSPORTER 7 PRECURSOR); cation transmembrane transporter/ metal ion transmembrane transporter/ zinc ion transmembrane transporter |
| AT4G33020 | 253413_at | ATZIP9__ZIP9; cation transmembrane transporter/ metal ion transmembrane transporter |
| AT1G31260 | 262546_at | ZIP10 (ZINC TRANSPORTER 10 PRECURSOR); cation transmembrane transporter/ metal ion transmembrane transporter |
| AT1G55910 | 260601_at | ZIP11 (ZINC TRANSPORTER 11 PRECURSOR); cation transmembrane transporter/ metal ion transmembrane transporter |
| AT5G62160 | 247456_at | AtZIP12 (ZINC TRANSPORTER 12 PRECURSOR); cation transmembrane transporter/ metal ion transmembrane transporter |
| AT2G46800 | 266718_at | ATMTP1_MTP1_ZAT1__ZAT (ZINC TRANSPORTER OF ARABIDOPSIS THALIANA); inorganic anion transmembrane transporter/ metal ion transmembrane transporter/ zinc ion transmembrane transporter |
| AT3G61940 | 251294_at | ATMTPA1__MTPA1; efflux transmembrane transporter/ inorganic anion transmembrane transporter/ zinc ion transmembrane transporter |
| AT3G58810 | 251545_at | ATMTPA2_MTP3__MTPA2 (METAL TOLERANCE PROTEIN A2); efflux transmembrane transporter/ inorganic anion transmembrane transporter/ zinc ion transmembrane transporter |
| AT2G29410 | 266273_at | ATMTPB1__MTPB1 (METAL TOLERANCE PROTEIN B1); efflux transmembrane transporter/ inorganic anion transmembrane transporter/ zinc ion transmembrane transporter |
| AT3G58060 | 251620_at | cation efflux family protein / metal tolerance protein, putative (MTPc3) |
| AT1G79520 | 262940_at | cation efflux family protein |
| AT1G16310 | 262751_at | cation efflux family protein |
| AT2G39450 | 266963_at | ATMTP11__MTP11; cation transmembrane transporter/ manganese ion transmembrane transporter/ manganese:hydrogen antiporter |
| AT2G47830 | 266505_at | cation efflux family protein / metal tolerance protein, putative (MTPc1) |
| AT3G12100 | 256272_at | cation efflux family protein / metal tolerance protein, putative |
| AT1G51610 | 260489_at | cation efflux family protein / metal tolerance protein, putative (MTPc4) |
| AT4G37270 | 246276_at | ATHMA1__HMA1; ATPase/ cadmium-transporting ATPase/ calcium-transporting ATPase/ copper-exporting ATPase/ zinc transporting ATPase |
| AT4G30110 | 253657_at | HMA2; cadmium-transporting ATPase |
| AT4G30120 | 253658_at | ATHMA3__HMA3 (HEAVY METAL ATPASE 3); ATPase, coupled to transmembrane movement of ions, phosphorylative mechanism |
| AT2G19110 | 267488_at | HMA4; cadmium ion transmembrane transporter/ cadmium-transporting ATPase/ zinc ion transmembrane transporter |
| AT1G63440 | 261551_at | HMA5 (HEAVY METAL ATPASE 5); ATPase, coupled to transmembrane movement of ions, phosphorylative mechanism |
| AT4G33520 | 253339_at 253342_at | HMA6__PAA1 (P-TYPE ATP-ASE 1); ATPase, coupled to transmembrane movement of ions, phosphorylative mechanism / copper ion transmembrane transporter |
| AT5G44790 | 249027_at | HMA7__RAN1 (RESPONSIVE-TO-ANTAGONIST 1); ATPase, coupled to transmembrane movement of ions, phosphorylative mechanism / copper ion transmembrane transporter |
| AT1G23020 | 264751_at | ATFRO3__FRO3; ferric-chelate reductase |
| AT5G49730 | 248566_s_at | FRO6__ATFRO6 (FERRIC REDUCTION OXIDASE 6); ferric-chelate reductase/ oxidoreductase |
| AT5G49740 | 248566_s_at | FRO7__ATFRO7 (FERRIC REDUCTION OXIDASE 7); ferric-chelate reductase/ oxidoreductase |
| AT5G50160 | 248540_at | ATFRO8__FRO8 (FERRIC REDUCTION OXIDASE 8); ferric-chelate reductase/ oxidoreductase |
| AT3G56980 | 251677_at | ORG3__BHLH039; DNA binding / transcription factor |
| AT5G04150 | 245692_at | BHLH101; DNA binding / transcription factor |
| AT1G56160 | 262091_at | ATMYB72__MYB72 (MYB DOMAIN PROTEIN 72); DNA binding / transcription factor |
| AT3G12820 | 257689_at | AtMYB10 (myb domain protein 10); DNA binding / transcription factor |
| AT4G09110 | 255075_at | zinc finger (C3HC4-type RING finger) family protein |
| AT3G13610 | 256647_at | oxidoreductase, 2OG-Fe(II) oxygenase family protein |
| AT5G06490 | 250730_at | zinc finger (C3HC4-type RING finger) family protein |
| AT2G20030 | 265582_at | zinc finger (C3HC4-type RING finger) family protein |
| AT3G08040 | 258646_at | MAN1__FRD3 (FERRIC REDUCTASE DEFECTIVE 3); antiporter/ transporter |
| AT2G01770 | 265863_at | VIT1 (vacuolar iron transporter 1); iron ion transmembrane transporter |
| AT5G03570 | 250952_at | ATIREG2 (IRON-REGULATED PROTEIN 2); nickel ion transmembrane transporter |
| AT2G38460 | 267029_at | ATIREG1 (IRON-REGULATED PROTEIN 1); transporter |
| AT5G26820 | 246847_at | ATIREG3 (IRON-REGULATED PROTEIN 3) |
| AT1G80830 | 261895_at | ATNRAMP1_PMIT1__NRAMP1 (NATURAL RESISTANCE-ASSOCIATED MACROPHAGE PROTEIN 1); inorganic anion transmembrane transporter/ manganese ion transmembrane transporter/ metal ion transmembrane transporter |
| AT1G47240 | 260509_at | ATNRAMP2__NRAMP2; inorganic anion transmembrane transporter/ metal ion transmembrane transporter |
| AT2G23150 | 267266_at | ATNRAMP3__NRAMP3 (NATURAL RESISTANCE-ASSOCIATED MACROPHAGE PROTEIN 3); inorganic anion transmembrane transporter/ manganese ion transmembrane transporter/ metal ion transmembrane transporter |
| AT5G67330 | 247001_at | NRAMP4__ATNRAMP4; inorganic anion transmembrane transporter/ manganese ion transmembrane transporter/ metal ion transmembrane transporter |
| AT4G18790 | 254640_at | ATNRAMP5__NRAMP5; inorganic anion transmembrane transporter/ metal ion transmembrane transporter |
| AT1G15960 | 261845_at | ATNRAMP6__NRAMP6; inorganic anion transmembrane transporter/ metal ion transmembrane transporter |
| AT3G46900 | 252502_at | COPT2; copper ion transmembrane transporter/ high affinity copper ion transmembrane transporter |
| AT5G20650 | 245999_at | COPT5; copper ion transmembrane transporter/ high affinity copper ion transmembrane transporter |
| AT5G59030 | 247745_at | COPT1 (copper transporter 1); copper ion transmembrane transporter |
| AT5G59040 | 247759_at | COPT3; copper ion transmembrane transporter/ high affinity copper ion transmembrane transporter |
| AT1G09240 | 264261_at | ATNAS3__NAS3 (NICOTIANAMINE SYNTHASE 3); nicotianamine synthase |
| AT1G56430 | 259632_at | NAS4 (NICOTIANAMINE SYNTHASE 4); nicotianamine synthase |
| AT5G04950 | 250832_at | ATNAS1__NAS1 (NICOTIANAMINE SYNTHASE 1); nicotianamine synthase |
| AT5G56080 | 248048_at | ATNAS2__NAS2 (NICOTIANAMINE SYNTHASE 2); nicotianamine synthase |
| AT1G07600 | 261438_at | ATMT-2_ATMT-Q_LSR4_MT-Q__MT1A (METALLOTHIONEIN 1A); copper ion binding / metal ion binding |
| AT2G23240 | 245070_at | plant EC metallothionein-like family 15 protein |
| AT2G42000 | 267579_at | plant EC metallothionein-like family 15 protein |
| AT3G09390 | 259008_at | ATMT-1_ATMT-K__MT2A (METALLOTHIONEIN 2A); copper ion binding |
| AT3G15353 | 257054_at | MT3 (METALLOTHIONEIN 3); copper ion binding |
| AT5G02380 | 250991_at | MT2B (METALLOTHIONEIN 2B); copper ion binding |
| AT5G44070 | 249078_at | ARA8_ATPCS1_PCS1__CAD1 (CADMIUM SENSITIVE 1); cadmium ion binding / copper ion binding / glutathione gamma-glutamylcysteinyltransferase |
| AT1G03980 | 265065_at | ATPCS2 (phytochelatin synthase 2); glutathione gamma-glutamylcysteinyltransferase |
| AT2G40300 | 263831_at | ATFER4 (ferritin 4); binding / ferric iron binding / oxidoreductase/ transition metal ion binding |
| AT3G56090 | 251735_at | ATFER3 (ferritin 3); binding / ferric iron binding / oxidoreductase/ transition metal ion binding |
| AT5G01600 | 251109_at | ATFER1; ferric iron binding / iron ion binding |
| AT3G11050 | 256416_at | ATFER2 (ferritin 2); binding / ferric iron binding / oxidoreductase/ transition metal ion binding |
| AT1G09560 | 264506_at | GLP5 (GERMIN-LIKE PROTEIN 5); manganese ion binding / nutrient reservoir |
| AT3G62020 | 251297_at | GLP10 (GERMIN-LIKE PROTEIN 10); manganese ion binding / nutrient reservoir |
| AT5G26700 | 246841_at | germin-like protein, putative |
| AT3G05930 | 258748_at | GLP8 (GERMIN-LIKE PROTEIN 8); manganese ion binding / nutrient reservoir |
| AT1G18980 | 259478_at | germin-like protein, putative |
| AT1G18970 | 259481_at | GLP4 (GERMIN-LIKE PROTEIN 4); manganese ion binding / nutrient reservoir |
| AT3G05950 | 258746_at | germin-like protein, putative |
| AT5G39110 | 249490_s_at | germin-like protein, putative |
| AT5G39150 | 249490_s_at | germin-like protein, putative |
| AT3G04200 | 258578_at | germin-like protein, putative |
| AT5G39100 | 249495_at | GLP6 (GERMIN-LIKE PROTEIN 6); manganese ion binding / nutrient reservoir |
| AT5G39180 | 249490_s_at | germin-like protein, putative |
| AT5G39120 | 249490_s_at | germin-like protein, putative |
| AT5G38910 | 249476_at | germin-like protein, putative |
| AT4G14630 | 245567_at | GLP9 (GERMIN-LIKE PROTEIN 9); manganese ion binding / nutrient reservoir |
| AT5G38940 | 249477_s_at | manganese ion binding / nutrient reservoir |
| AT5G38930 | 249477_s_at | germin-like protein, putative |
| AT5G39160 | 249474_s_at | germin-like protein (GLP2a) (GLP5a) |
| AT5G39190 | 249474_s_at | ATGER2_GLP2A__GER2 (GERMIN-LIKE PROTEIN 2); oxalate oxidase |
| AT5G38960 | 249479_at | germin-like protein, putative |
| AT5G39130 | 249474_s_at 249491_at | germin-like protein, putative |
| AT3G62010 | 251296_at | unknown protein |
| AT3G04150 | 258582_at | germin-like protein, putative |
| AT5G39160 | 249474_s_at | germin-like protein (GLP2a) (GLP5a) |
| AT3G04190 | 258579_s_at | germin-like protein, putative |
| AT3G04180 | 258579_s_at | germin-like protein, putative |
| AT3G04170 | 258580_at | germin-like protein, putative |
| AT3G10080 | 258938_at | germin-like protein, putative |
| AT5G26710 | 246845_at | glutamate-tRNA ligase, putative / glutamyl-tRNA synthetase, putatuve / GluRS, putative |
| AT1G10460 | 263211_at | GLP7 (GERMIN-LIKE PROTEIN 7); manganese ion binding / nutrient reservoir |
| AT1G74820 | 262214_at | cupin family protein |
| AT5G20630 | 246004_at | ATGER3_GLP3_GLP3A_GLP3B__GER3 (GERMIN 3); oxalate oxidase |
| AT1G72610 | 259892_at | ATGER1_GLP1__GER1 (GERMIN-LIKE PROTEIN 1); oxalate oxidase |
| AT5G61750 | 247516_at | cupin family protein |
| AT5G63530 | 247331_at | ATFP3; metal ion binding / transition metal ion binding |
| AT5G55930 | 248037_at | ATOPT1__OPT1 (OLIGOPEPTIDE TRANSPORTER 1); oligopeptide transporter |
| AT1G09930 | 264659_at | ATOPT2; oligopeptide transporter |
| AT4G16370 | 245296_at | OPT3__ATOPT3 (OLIGOPEPTIDE TRANSPORTER); oligopeptide transporter |
| AT5G64410 | 247284_at | ATOPT4__OPT4 (OLIGOPEPTIDE TRANSPORTER 4); oligopeptide transporter |
| AT4G26590 | 253984_at | ATOPT5__OPT5 (OLIGOPEPTIDE TRANSPORTER 5); oligopeptide transporter |
| AT4G10770 | 254938_at | ATOPT7__OPT7 (OLIGOPEPTIDE TRANSPORTER 7); oligopeptide transporter |
| AT5G53520 | 248275_at | OPT8__ATOPT8 (ARABIDOPSIS THALIANA OLIGOPEPTIDE TRANSPORTER 8); oligopeptide transporter |
| AT5G53510 | 248274_at | OPT9__ATOPT9 (ARABIDOPSIS THALIANA OLIGOPEPTIDE TRANSPORTER 9); oligopeptide transporter |
| AT1G60960 | 259723_at | IRT3; cation transmembrane transporter/ metal ion transmembrane transporter |
| AT5G53450 | 248270_at | ORG1 (OBP3-responsive gene 1); ATP binding / kinase/ protein kinase |
| AT3G55370 | 251806_at | OBP3 (OBF-BINDING PROTEIN 3); DNA binding / transcription factor |
| AT5G38820 | 249535_at | amino acid transporter family protein |
| AT4G30120 | 253658_at | ATHMA3__HMA3 (HEAVY METAL ATPASE 3); ATPase, coupled to transmembrane movement of ions, phosphorylative mechanism |
| AT1G34760 | 262412_at | GF14 OMICRON__GRF11 (GENERAL REGULATORY FACTOR 11); ATPase binding / amino acid binding / protein binding / protein phosphorylated amino acid binding |
| AT4G31940 | 253502_at | CYP82C4; electron carrier/ heme binding / iron ion binding / monooxygenase/ oxygen binding |
| AT3G53280 | 251987_at | CYP71B5 (CYTOCHROME P450 71B5); electron carrier/ heme binding / iron ion binding / monooxygenase/ oxygen binding |
| AT5G02780 | 250983_at | In2-1 protein, putative |
| AT3G12900 | 257135_at | oxidoreductase, 2OG-Fe(II) oxygenase family protein |
| AT3G11750 | 258782_at | dihydroneopterin aldolase, putative |
| AT4G02330 | 255524_at | ATPMEPCRB; pectinesterase |
| AT5G36890 | 249636_at | BGLU42 (BETA GLUCOSIDASE 42); beta-glucosidase/ catalytic/ cation binding / hydrolase, hydrolyzing O-glycosyl compounds |
| AT3G58060 | 251620_at | cation efflux family protein / metal tolerance protein, putative (MTPc3) |
| AT3G51200 | 252145_at | auxin-responsive family protein |
| AT1G14190 | 262655_s_at | glucose-methanol-choline (GMC) oxidoreductase family protein |
| AT3G50740 | 252183_at | UGT72E1 (UDP-glucosyl transferase 72E1); UDP-glycosyltransferase/ coniferyl-alcohol glucosyltransferase/ transferase, transferring glycosyl groups |
| AT4G05030 | 255254_at | heavy-metal-associated domain-containing protein |
| AT3G01550 | 259185_at | PPT2 (PHOSPHOENOLPYRUVATE (PEP)/PHOSPHATE TRANSLOCATOR 2); antiporter/ triose-phosphate transmembrane transporter |
| AT1G44130 | 245738_at | nucellin protein, putative |
| AT5G07880 | 250551_at | ATSNAP29__SNAP29; SNAP receptor |
| AT3G60420 | 251400_at | LOCATED IN: cellular_component unknown; EXPRESSED IN: 17 plant structures; EXPRESSED DURING: 10 growth stages; CONTAINS InterPro DOMAIN/s: Phosphoglycerate mutase (InterPro:IPR013078); BEST Arabidopsis thaliana protein match is: unknown protein (TAIR:AT3G60450.1); Has 196 Blast hits to 165 proteins in 65 species: Archae - 0; Bacteria - 41; Metazoa - 2; Fungi - 34; Plants - 58; Viruses - 0; Other Eukaryotes - 61 (source: NCBI BLink). |
| AT2G30670 | 267573_at | tropinone reductase, putative / tropine dehydrogenase, putative |
| AT4G08800 | 255112_at | protein kinase, putative |
| AT3G56980 | 251677_at | ORG3__BHLH039; DNA binding / transcription factor |
| AT3G55670 | 251747_at | FUNCTIONS IN: molecular_function unknown; INVOLVED IN: biological_process unknown; LOCATED IN: cellular_component unknown; CONTAINS InterPro DOMAIN/s: FBD (InterPro:IPR013596), FBD-like (InterPro:IPR006566); BEST Arabidopsis thaliana protein match is: F-box family protein (TAIR:AT3G52680.2); Has 274 Blast hits to 168 proteins in 2 species: Archae - 0; Bacteria - 0; Metazoa - 0; Fungi - 0; Plants - 274; Viruses - 0; Other Eukaryotes - 0 (source: NCBI BLink). |
| AT1G26420 | 261005_at | FAD-binding domain-containing protein |
| AT5G13320 | 250286_at | GDG1_WIN3_WIN3__PBS3 (AVRPPHB SUSCEPTIBLE 3) |
| AT4G31970 | 253505_at | CYP82C2; electron carrier/ heme binding / iron ion binding / monooxygenase/ oxygen binding |
| AT4G33710 | 253352_at | pathogenesis-related protein, putative |
| AT2G15290 | 263298_at | ATTIC21_CIA5_PIC1__TIC21 (TRANSLOCON AT INNER MEMBRANE OF CHLOROPLASTS 21); copper uptake transmembrane transporter/ iron ion transmembrane transporter/ protein homodimerization |
| AT2G15290 | 263298_at | ATTIC21_CIA5_PIC1__TIC21 (TRANSLOCON AT INNER MEMBRANE OF CHLOROPLASTS 21); copper uptake transmembrane transporter/ iron ion transmembrane transporter/ protein homodimerization |
